# Interaction of Pubertal Development and Metabolic Control in Adolescents with Type 1 Diabetes Mellitus

**DOI:** 10.1155/2017/8615769

**Published:** 2017-11-07

**Authors:** M. Plamper, B. Gohlke, J. Woelfle, K. Konrad, T. Rohrer, S. Hofer, W. Bonfig, K. Fink, R. W. Holl

**Affiliations:** ^1^Pediatric Endocrinology Division, Children's Hospital, University of Bonn, Bonn, Germany; ^2^Department of Pediatrics and Adolescent Medicine, University of Cologne, Cologne, Germany; ^3^Department of Pediatric and Adolescent Medicine, Elisabeth Hospital Essen, Essen, Germany; ^4^Department of Pediatrics, University of Saarland, Homburg, Germany; ^5^Department of Pediatrics, University of Innsbruck, Innsbruck, Austria; ^6^Department of Pediatrics, Technical University Munich, Munich, Germany; ^7^Department of Pediatrics, Klinikum Wels-Grieskirchen, Wels, Austria; ^8^Institute for Epidemiology and Medical Biometry, ZIBMT University of Ulm and German Center for Diabetes Research (DZD), Neuherberg, München, Germany

## Abstract

**Background:**

In T1DM, delayed pubertal development and reduced final height are associated with inadequate metabolic control.

**Objective:**

To assess whether T1DM affects pubertal growth spurt and whether metabolic control during puberty is gender-related.

**Methods:**

Using a large multicentre database, longitudinal data from 1294 patients were analysed. Inclusion criteria: complete records of height and HbA1c from the age of seven to 16 years. Exclusion criteria: other significant chronic diseases and medications, T1DM duration less than three months, and initial BMI < 3rd or >97th percentile.

**Results:**

Growth velocity (GV) was impaired with a significant reduction of peak GV by 1.2 cm in boys. HbA1c increase during male puberty was lower except for a period of 1.5 years. The highest HbA1c increase in boys coincided with maximum growth spurt. In girls, the highest HbA1c increase was observed during late puberty. Even though there is impaired GV, both sexes reach a height at 16 years of age which corresponds to the background population height.

**Conclusion:**

Worsening of metabolic control is sex-discordant and associated with gender-specific alterations of GV. However, the vast majority of boys and girls with T1DM seems to reach normal height at the age of 16 years.

## 1. Introduction

Type 1 diabetes mellitus (T1DM) is a chronic disease frequently manifesting in childhood or adolescence. Like other chronic diseases, T1DM can have a negative impact on growth and pubertal development. Several studies describe a delayed onset of puberty in both sexes and a delay of menarche in girls with diabetes [1–4].

Although the therapeutic regimen in children and adolescents has changed over the years towards a diversified arsenal of differently acting insulin types and a larger fraction of patients treated with CSII, adult height remains still reduced in patients with T1DM in the 21st century [5, 6].

Metabolic control in T1DM frequently worsens during puberty [7–9]. In this context, puberty-related insulin resistance may play an important role; in addition, inadequate adherence to diabetes management seems to be involved [9].

In this study, we analysed the relationship between pubertal growth spurt and HbA1c deterioration. Furthermore, we analysed whether differences in metabolic control are sex-specific. Since adult height is impaired in children with T1DM, we speculated that an impaired pubertal growth spurt might be directly associated with insufficient metabolic control. We therefore compared timing and degree of peripubertal growth velocity of our patients in relation to metabolic control with German reference data [10].

## 2. Methods

DPV (Diabetes Patienten Verlaufsdaten) is a diabetes registry for quality management and research that started in 1995. Approximately 325 paediatric diabetes centres in Germany and Austria participate in DPV. The participating centres enter their routine care data into an electronic health record; anonymized data are analysed every six months.

The inclusion criteria for this study were a continuous recording of height and HbA1c at least every six months from the age of seven to 16 years in patients with T1DM.

Patients with celiac disease, TSH > 10 *μ*U/ml, eating disorders, and glucocorticoid or growth hormone therapy were excluded. Also, patients with duration of diabetes less than three months and patients with a BMI below the 3rd or above the 97th percentile at the age of seven years were excluded. A different quality of metabolic control during the first year of disease might influence growth and weight and may therefore not be compared to those who have suffered from type 1 diabetes for a longer period of time. We therefore added an analysis, comparing whether height velocity in patients with duration of 3–12 months differs from those with a duration exceeding 12 months and found no significant difference in height velocity between the groups.

In the DPV database, 9920 patients had diabetes onset before 7.5 years of age and were older than 15.5 years at the time of analysis. Applying all exclusion criteria, 1294 patients with a complete longitudinal dataset could be analysed (male *n* = 664, female *n* = 630). Clinical characteristics of these patients are depicted in Table 1.

HbA1c levels were determined using the methodology established at each centre. To adjust for differences among laboratories, the HbA1c values were mathematically standardized to the DCCT (Diabetes Control and Complications Trial) with a reference range of 4.05% to 6.05% [11] using the multiple-of-the-mean method.

To quantify the deterioration of HbA1c, we calculated the change of HbA1c in individual patients between two consecutive time points for every year (Δ HbA1c = (mean HbA1c age (*n* + 1)) − (mean HbA1c age (*n*)). We used SAS version 9.3 for statistical analysis. *p* values were based on a general linear model. We compared the growth velocity data of our patients with data from a healthy control group surveyed by Brandt and Reinken [10] and used *t*-tests to assess for statistical significance.

Although there are newer reference data in German children available, we decided to use the reference data from Brandt and Reinken, since these in contrast to the more recent growth data had been collected longitudinally. Because body mass index (BMI) is not normally distributed, we used the LMS method to calculate SDS-BMI as a measure for the degree of overweight. The LMS method was chosen as it summarizes the data in terms of three smooth age-specific curves called lambda (L), mu (M), and sigma (S) based on German population-specific data [12]. The M and S curves, respectively, correspond to the median and coefficients of variation (CV) of BMI for German children at each age and gender, whereas L allows for the substantial age-dependent skewness in the distribution of BMI [11, 13]. The assumption underlying the LMS method is that after Box-Cox power transformation, the BMI data at each age are normally distributed [11].

## 3. Results

### 3.1. Metabolic Control

**Figure 1:**
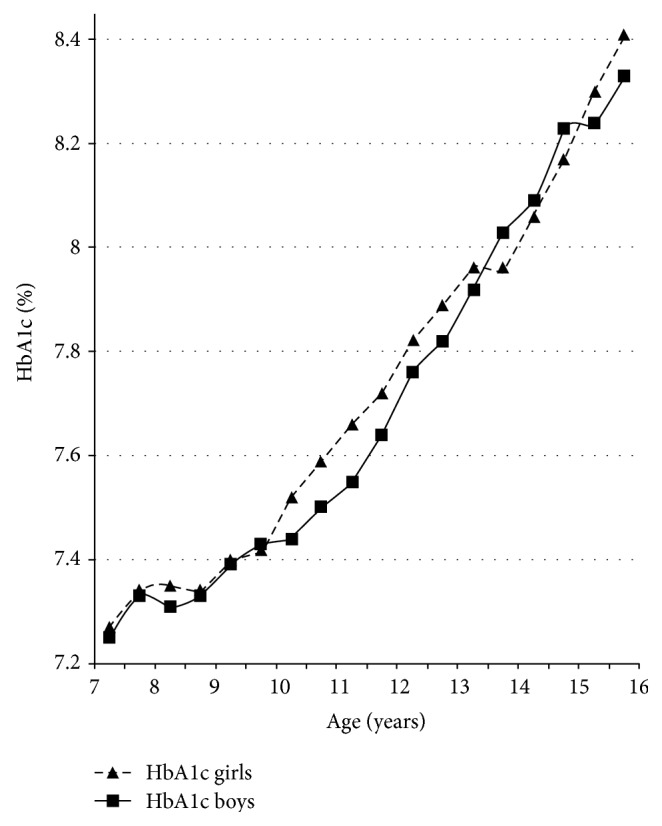
Gender-specific course of HbA1c concentration before and during puberty.

Mean HbA1c levels increased continuously from 7.3% (56.3 mmol/mol) at the age of seven years to 8.4% (68.3 mmol/mol) at the age of 16 years (Figure 1 and Table 2). Peak HbA1c in girls (9.37 [9.25; 9.50]) was significantly higher than peak HbA1c in boys (9.17 [9.05; 9.29]) (delta 0.20 [0.03; 0.37], *p* = 0.02). From 10 years onwards, mean HbA1c was always lower in boys as compared to girls except for a period of 1.5 years between 13.5 and 15 years of age.

The highest HbA1c increase in boys (increment to preceding measurement + 0.4, *p* = 0.027) occurred between 12 and 14 years, which covered the time of usual maximum growth spurt at the age of 13 to 14 years.

**Figure 2:**
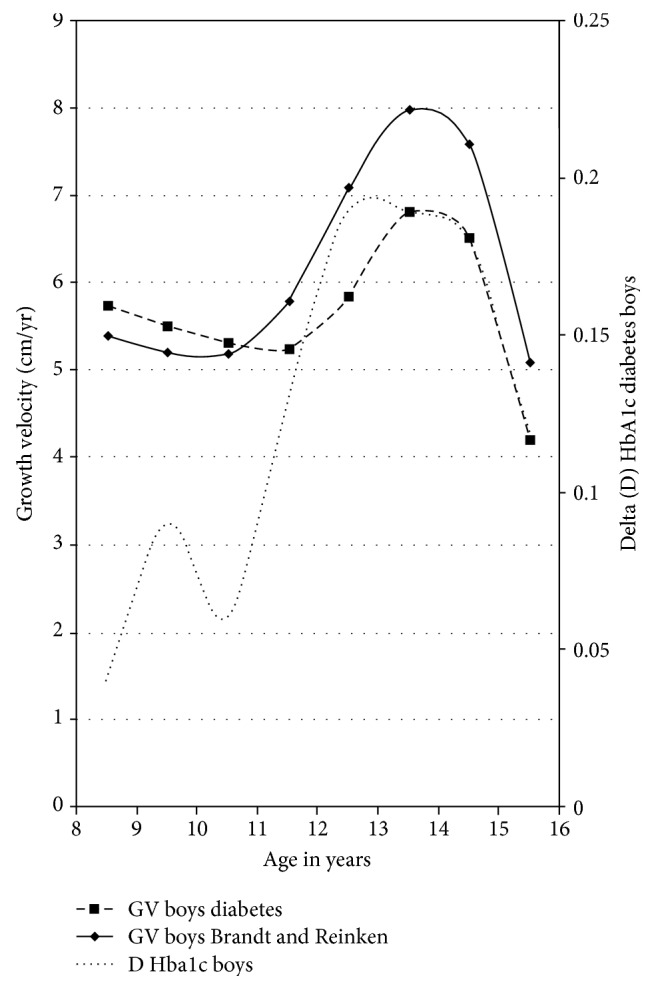
Growth velocity and delta HbA1c in boys with T1DM.

**Figure 3:**
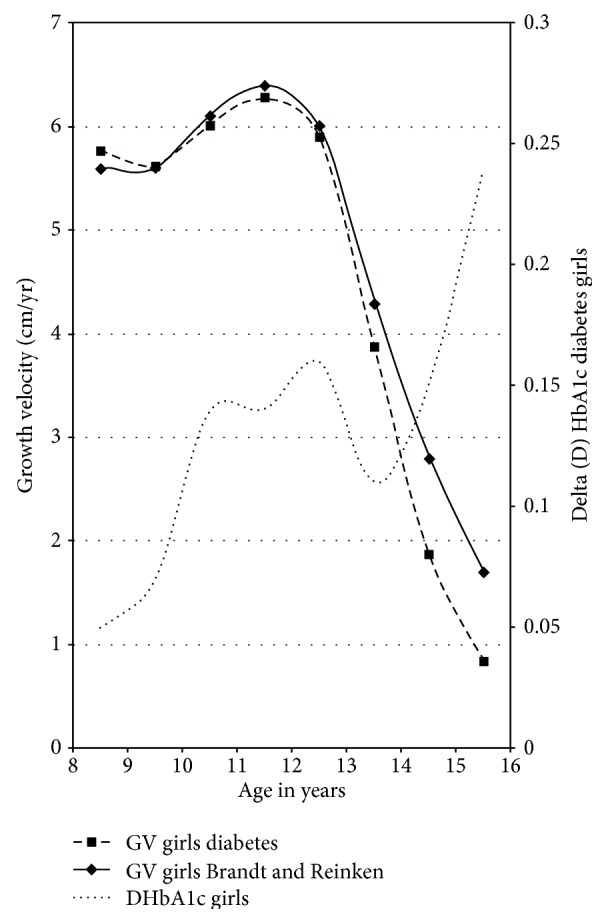
Growth velocity and delta HbA1c in girls with T1DM.

Girls exhibited their maximum growth spurt on average between 11 and 12 years of age. In girls, we found a significantly higher HbA1c increase between 10 and 11 years (increment to preceding measurement + 0.14, *p* = 0.012) and between 15 and 16 years (increment to preceding measurement + 0.2, *p* = 0.018) (Table 2). Thus, in girls, HbA1c increase was not strictly related to the time of maximal growth spurt (Figures 2 and 3).

### 3.2. Growth Velocity

We compared growth velocities of diabetes patients with growth velocity data from a healthy control group published by Brandt and Reinken [10]. We started to analyse and to compare growth velocity of our diabetes patients between the ages of eight and nine years with the data published by Brandt and Reinken.

This time interval from the start of the observation period was chosen to make sure that all patients were most likely to have finished remission and had made enough experience with diabetes management to exclude a respective influence on the quality of diabetes control.

**Figure 4:**
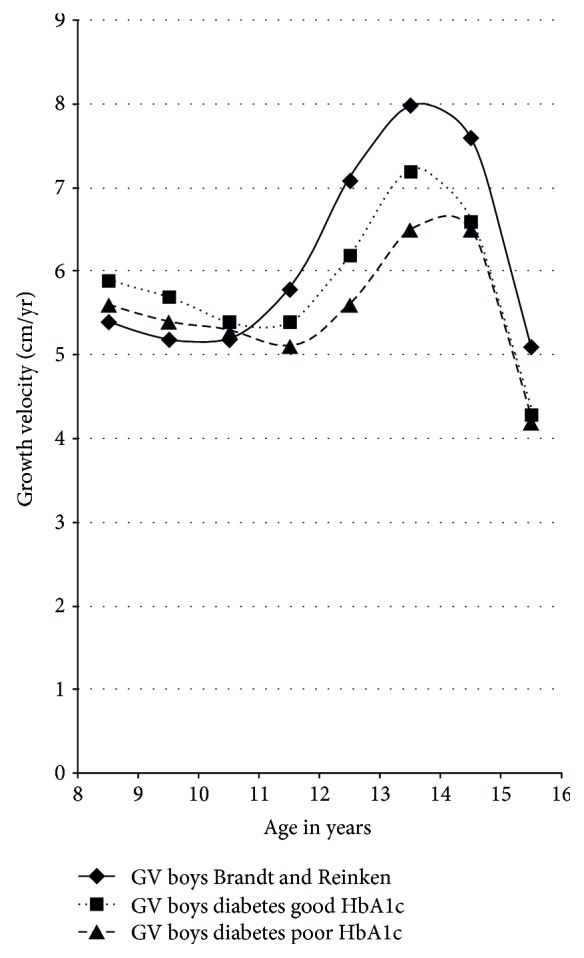
Growth velocity before and during puberty in boys with T1DM versus reference population.

**Figure 5:**
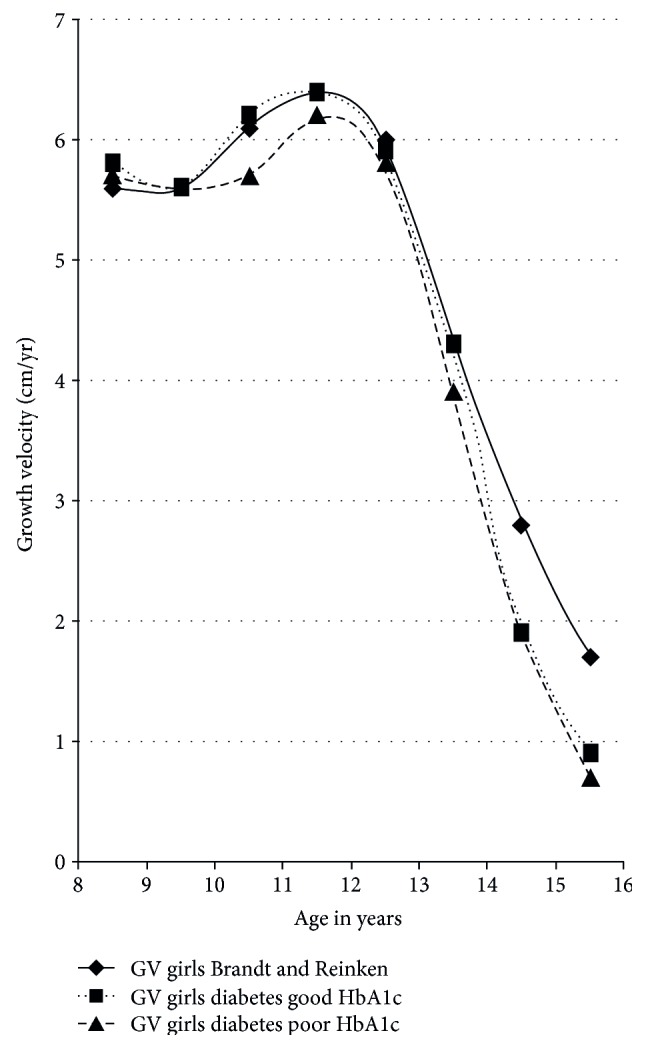
Growth velocity before and during puberty in girls with T1DM versus reference population.

Timing of pubertal growth spurt in girls and boys with T1DM did not differ from the healthy control population. However, in boys with T1DM, median growth velocity at the time of maximal growth spurt was significantly reduced by 1.2 cm compared to the data of Brandt and Reinken (*p* < 0.001; Figure 4). In girls, we found no difference in median maximum growth velocity (*p* = 0.5), but growth velocity declined more rapidly in diabetic girls after reaching pubertal peak height velocity compared to the reference population, as visualized in Figure 5. To compare patients who had adequate to those who had inadequate metabolic control, the respective groups were stratified by HbA1c. The group with good metabolic control was defined by a long-term mean HbA1c < 7.5% (*n* = 647, female *n* = 308, male *n* = 340), and the group with poor metabolic control had a mean HbA1c equal or higher than 7.5% over all the years of documentation (*n* = 647, female *n* = 323, male *n* = 324).

The classification into two groups (good metabolic control and poor metabolic control) was based on medium HbA1c throughout all observation years, including six-month intervals. The median HbA1c of 7592 was chosen to divide our patients' group into two equally sized groups.

Compared to healthy children, growth spurt was impaired in both groups, but the impairment was more pronounced in patients with poor metabolic control (Figures 4 and 5).

To have a closer view on those with poor metabolic control, we subdivided this group into two parts: one part with poor metabolic control (HbA1c > 7.5%) (*n* = 537) and the other part with very poor metabolic control with a mean HbA1c > 9% (*n* = 110). As expected, the worst growth velocity was seen in those with a mean HbA1c > 9% (Table 3).

### 3.3. Height SDS

At the start of the age of seven years, diabetic girls and boys had reached a height SDS of +0.17 (interquartile range −0.45 to +0.85) compared to German reference data [12]. For both boys and girls with T1DM, height SDS during the observation period differed significantly from the reference population (*p* < 0.0001, resp.). However, at the end of this study at the age of 16 years, height SDS of both boys and girls was no longer significantly different from reference data (height SDS in girls + 0.04, interquartile range −0.65 to +0.69, n.s; height SDS in boys – 0.02, interquartile range −0.64 to 0.62, n.s.).

### 3.4. BMI-SDS

**Figure 6:**
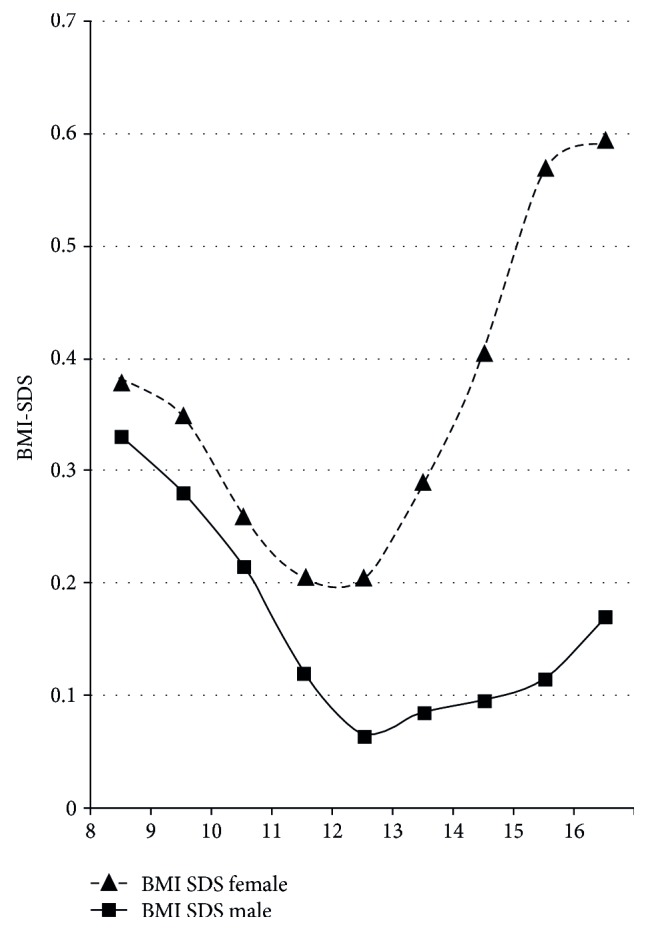
Gender-specific course of BMI-SDS before and during puberty.

During the whole period from seven to 16 years of age, BMI-SDS of our patients was higher than the mean BMI-SDS of German reference data [13]. BMI-SDS declined from the age of seven until the age of 11.5 years but increased continuously thereafter (Figure 6). Girls with T1DM had a higher absolute mean BMI-SDS than boys with T1DM. This difference became increasingly obvious after the age of ten years.

By the age of 16 years, the mean BMI-SDS of girls was +0.61 (interquartile range + 0.12 to 1.06), and BMI-SDS of boys was + 0.18 (interquartile range −0.40 to +0.68). The median BMI (50th percentile) of 16-year-old girls in our cohort with T1DM was 23.4 kg/m^2^ and corresponded to a BMI between the 75th and 90th percentile (exactly corresponding to the 81st percentile) of German reference data [12]. In 16-year-old boys, the median BMI of 21.5 kg/m^2^ corresponded to a BMI between the 50th and 75th percentile (exactly corresponding to the 64th percentile) of reference data. For both boys and girls with T1DM, BMI-SDS differed significantly from the reference population (*p* < 0.0001, resp.) throughout the study period (*p* < 0.0001, resp.) and at the end of this study at an age of 16 years (*p* < 0.0001, resp.).

Interestingly, girls and boys with a satisfactory metabolic control exhibited a lower BMI-SDS than those with a mean HbA1c > 7.5% over the observation period. 16-year-old girls with adequate metabolic control had a BMI-SDS of +0.5 (interquartile range −0.05 to 1.0) versus +0.67 (interquartile range 0.25 to 1.13) in girls with poor metabolic control. In 16-year-old boys, the BMI-SDS was +0.15 (interquartile range −0.43 to 0.64) in those with good metabolic control versus +0.24 (interquartile range −0.37 to 0.69) in those with poor metabolic control.

### 3.5. Self-Monitoring of Blood Glucose (SMBG)

Documented blood glucose self-monitoring frequency was not significantly different between girls and boys. The reported frequency of all patients, independent of treatment regimes, increased from the age of seven (mean 4.97 measurements per day) to 12 (mean 5.43 measurements per day) and declined afterwards. At 16 years of age, the reported frequency was not different from that with seven years of age. The mean SMBG over the whole period of time was 5.2 measurements a day. Girls and boys with a good mean HbA1c had a higher SMBG (arithmetical mean good HbA1c versus poor HbA1c: 5.5 versus 4.95 SMBG) than those with an inadequate metabolic control. In the course of the data collection and passing time, the percentage of patients using an insulin pump treatment increased from 1% to 36%.

Those patients who were using an insulin pump exhibited a higher frequency of SMBG in all age groups compared to patients using ICT. In both groups (ICT and CSII), the SMBG frequency declined with age. At the age of seven years, the mean SMBG was 4.97 for ICT versus 6.52 SMBG with insulin pump treatment. At the age of sixteen years, SMBG was 4.77 (ICT) versus 5.36 (CSII). Taken together, SMBG frequency in this cohort was influenced by age, time period, and mode of insulin treatment.

## 4. Discussion

Adherence to diabetes therapy and consecutive metabolic control in adolescent type 1 diabetes is challenging. Furthermore, diabetes control in puberty is modulated by a change in synthesis and secretion of several hormones, including growth hormone, which associate with changes in insulin sensitivity. In this longitudinal study, we found significant gender differences regarding metabolic control during puberty and marked differences with regard to gender-specific growth patterns.

Pubertal growth is characterized by the so-called pubertal growth spurt with a puberty-related increase of growth velocity. Change of growth velocity is a reliable sign of puberty [14]. In our study, we found that timing of pubertal growth spurt in girls and boys with T1DM did not differ from the healthy control described by Brandt and Reinken. However, mean growth velocity in diabetic boys at the time of maximal growth spurt was significantly reduced by 1.2 cm/year compared to that in healthy boys. In girls, we found no difference in median maximal growth velocity, which might be due to the relatively flat female pubertal growth spurt in comparison to the more pronounced male pubertal growth spurt. However, in girls with T1DM, growth velocity declined significantly faster after reaching peak growth velocity than in the reference population.

The reduced growth velocity in puberty can explain the loss of height during the course of diabetes [5]. Boys seem to lose height at the time of maximum growth spurt, whereas girls seem to lose height because of a more rapid decline in growth velocity afterwards. Nevertheless, it is rather relieving that height at the age of 16 years seems to be at exactly the same level like background population height, even though the height at the age of seven years lies above average (SDS + 0.17). Whether the taller height at the age of seven reflects differences in the growth pattern of diabetic subjects due to altered nutrient intake and metabolic control remains a subject of speculation.

Several studies with small patient numbers tried to analyse growth data in patients with T1DM. Salerno et al. [6] followed 62 subjects with T1DM from the onset of the disease until final height. At diagnosis, height was above the genetic target height, but in the following years, they saw, similar to our results, a reduced height gain. The authors were not able to explain these findings, as it seemed to be independent of the duration of T1DM or of metabolic control. Ahmed et al. [15] examined 46 children with T1DM. Height was measured every three months, and bone age was assessed annually. In addition, HbA1c, IGF-I, and C-peptide were analysed. Similar to our study, the timing of peak height velocity was in the normal range in both sexes. However, in contrast to our study, maximum growth spurt was reduced in girls, but not in boys. In their study, bone age of girls with T1DM was advanced at the onset of puberty, potentially explaining the different findings. In a cohort of 72 Sudanese diabetic children who were treated with a conventional insulin regime using purified bovine insulin, growth velocity between diagnosis and final height was slow with a significant reduction in pubertal growth spurt [16]. The metabolic control of this cohort was rather poor with a mean HbA1c level of 9.8%. As expected, adolescents with poor metabolic control showed a more impaired growth velocity compared to patients with a satisfactory mean HbA1c over the whole period of time.

In accordance with others, we found an increase in mean HbA1c levels during puberty, which were clearly above target HbA1c levels recommended in current guidelines [7, 8, 17, 18].

In contrast to most other studies, concerning growth in diabetes, metabolic control in our collective was still quite good [16].

For nearly the whole period of puberty from ten to 16 years of age, boys had a lower mean HbA1c concentration than girls. This is in accordance with the majority of other studies demonstrating a sex difference with worse metabolic control in females [4, 19–22]. Only a few studies were not able to find differences in metabolic control between men and women [7, 15]. Indeed, for a short interval of 1.5 years, our data showed a higher mean HbA1c concentration in boys compared to diabetic girls. Interestingly, this covered the period of maximum male pubertal growth spurt. Puberty and especially pubertal growth spurt are triggered by an interaction of gonadotropic (GnRH-LH/FSH-sex steroid) and somatotropic (GH-IGF-1) hormone axes [23]. Estrogens lead to an increase in the amplitude of pulsatile GH secretion; GH opposes insulin effects on glucose metabolism with GH overexposure typically resulting in impaired glucose homeostatic control. Androgens play a major role in the dramatic change in linear growth during puberty [24]. It is a well-known phenomenon that the increase of growth velocity is more pronounced in boys than in girls; this may be a consequence of higher GH amplitude in males compared to females. Furthermore, this increase in GH secretion might be responsible for a more pronounced dawn phenomenon in boys and therefore might underlie a deterioration of metabolic control around the maximum growth spurt. In contrast to boys, in our study, the highest mean HbA1c increase in girls was not associated with pubertal growth spurt but took place after the main hormonal changes characterizing female puberty. Hormonal differences [4] and different coping with a chronic disease may therefore play a role in the observed gender differences in glucose control during puberty [22]. Other causative factors for higher mean HbA1c concentration in females could be the higher prevalence of eating disorders and insulin purging, which might be of higher importance regarding growth as compared to a temporary impaired metabolic control [25–27].

Hoey and the Hvidoere study group could show that the quality of life (QoL) plays an important role in achieving a good metabolic control and that this score is typically worse in girls [28].

In addition, worse metabolic control in females could be modulated by the gender difference in weight development. The girls included in our cohort showed a significantly higher mean BMI-SDS than boys, especially at the age of 15-16 years, with a higher risk to become obese [13]. Higher BMI-SDS in adolescents with T1DM was described earlier [7, 29] and could have a negative influence on insulin sensitivity. This is supported by another study demonstrating greater insulin resistance in girls than boys during puberty [30].

In addition, hormonal changes and increasing insulin resistance during puberty [25, 31], treatment adherence, and family dynamics significantly influence metabolic control and HbA1c [28, 32]. A previous analysis of DPV data from 1995–2006 showed that self-monitoring of blood glucose (SMBG) declined with age and was lowest in children aged > 12 years. Additional SMBG up to five measurements per day improved metabolic control (HbA1c) especially in adolescents [33]. Our data underline that a higher frequency of SMBG is associated with a better HbA1c. Still, the differences were small and not statistically significant. Since DPV uses patient-reported SMBG and relies on the correctness of the patients' report, we cannot exclude that patients with worse metabolic control incorrectly answered the SMBG frequency.

Our study has several limitations: (a) target height of most patients was not available since this is not part of the routine data collection. (b) Documentation of Tanner stages was available only in 13% and was thus not included in our analysis. (c) Due to strict requirement of a sufficient auxological dataset in DPV, a large number of patients documented in DPV were not suitable and had to be excluded. However, patients that had to be excluded due to lack of auxological data did not differ regarding diabetes duration, therapy, or diabetes control. Still, the patient number of our study still remains significantly larger than that of all previous reports. (d) Since our population had its diabetes manifestation at the age of seven or younger, it is a specific group of diabetes patients. Growth patterns of patients who were older at the onset of the disease may be different. (e) In this study, we used relatively old reference data, since these in contrast to the more recent growth data had been collected longitudinally. We thus cannot exclude that this might have an impact on the interpretation of height data at the age sixteen years, although in Germany in recent decades there has been only a minor secular trend in height.

In conclusion, we found only a minor albeit significant affection of the growth pattern in diabetic subjects compared to the reference population. Furthermore, we detected that gender-discordant metabolic impairment during puberty, which in males, but not in females, correlated with peak growth velocity. The discrepancy that growth velocity in boys was more impaired than in girls even though girls' metabolic control was even worse over the whole period of time needs further investigations.

Thus, the care of patients with T1DM during puberty requires an enormous effort from the patient, the family, and the health care team, not only to deal with the psychological challenges of a chronic disease during the difficult period of puberty but also to improve the puberty-related risks for a poor metabolic control, reduced growth, and delayed pubertal development, although the effects on growth seem to be small.

## Figures and Tables

**Table 1:** Clinical characteristics of 1294 type 1 diabetic patients, depending on sex and metabolic control (data expressed as median and interquartile range).

	All	Female	Male	HbA1c < 7.5%	HbA1c > 7.5%
*n*	1294	630	664	647	647
Age at study start (yr)	7.25 (7.21; 7.29)	7.25 (7.22; 7.29)	7.25 (7.21; 7.29)	7.25 (7.21; 7.29)	7.25 (7.22; 7.29)
Age at end of study (yr)	15.75 (15.7; 15.79)	15.74 (15.69; 15.79)	15.75 (15.7; 15.79)	15.75 (15.7; 15.78)	15.74 (15.69; 15.79)
Diabetes duration at study start (yr)	2.56 (1.23; 4.09)	2.46 (1.15; 4.08)	2.63 (1.30; 4.15)	2.40 (1.22; 3.91)	2.84 (1.26; 4.29)
Diabetes duration at study end (yr)	11.07 (9.7; 12.58)	10.94 (9.66; 12.57)	11.13 (9.78; 12.61)	10.9 (9.67; 12.37)	11.3 (9.71; 12.79)
Height SDS at study start	0.17 (−0.45; 0.85)	0.17 (−0.43; 0.84)	0.17 (−0.45; 0.85)	0.25 (−0.32; 0.92)	0.12 (−0.51; 0.75)
Height SDS at study end	0.01 (−0.64; 0.65)	0.04 (−0.65; 0.69)	−0.02 (−0.64; 0.62)	0.19 (−0.44; 0.80)	−0.18 (−0.79; 0.48)
BMI-SDS at study start	0.37 (−0.18; 0.82)	0.38 (−0.15; 0.84)	0.35 (−0.21; 0.78)	0.36 (−0.20; 0.82)	0.39 (−0.17; 0.82)
BMI-SDS at study end	0.39 (−0.16; 0.89)	0.61 (0.12; 1.06)	0.18 (−0.40; 0.68)	0.30 (−0.24; 0.86)	0.46 (−0.07; 0.92)
HbA1c at study start (%)	7.17 (6.51; 7.89)	7.17 (6.51; 7.92)	7.15 (6.51; 7.88)	6.77 (6.22; 7.20)	7.72 (7.13; 8.32)
HbA1c at study end (%)	8.08 (7.33; 9.09)	8.12 (7.33; 9.19)	8.04 (7.33; 8.99)	7.42 (6.87; 7.98)	8.94 (8.18; 9.92)

**Table 2:** Gender-specific HbA1c development in patients with T1DM before and during puberty. Change in HbA1c (%) was calculated by the difference of the HbA1c concentration between the actual and the preceding year.

Age (years)	Δ HbA1c boys	Δ HbA1c girls	*p* value (Δ HbA1c boys versus girls)
8-9	0.04	0.05	0.05
9-10	0.09	0.07	0.63
10-11	0.06	**0.14**	**0.012**
11-12	0.13	0.14	0.065
12-13	0.19	0.16	0.023
13-14	0.19	0.11	0.31
14-15	0.18	0.15	0.23
15-16	0.12	**0.24**	**0.018**

**Table 3:** Growth velocity data of the whole dataset divided into good metabolic control (HbA1c < 7.5%), poor metabolic control (HbA1c > 7.5), and very poor metabolic control (HbA1c > 9%).

	Good metabolic control (HbA1c < 7.5%)	Poor metabolic control (HbA1c > 7.5%)	Very poor metabolic control (HbA1c > 9%)
*n*	647	537	110
Mean	5.38 (3.77; 7.02)	5.24 (3.63; 6.9)	4.86 (3.26; 6.46)
